# Serum Lipid Profile as a Predictor of Recurrence in Chronic Suppurative Otitis Media: A Retrospective Electronic Health Record-Based Cohort Study

**DOI:** 10.7759/cureus.114042

**Published:** 2026-08-06

**Authors:** Md. Masum Billah, Farzana Binte Abedin Leera

**Affiliations:** 1 Department of Otolaryngology, Head and Neck Surgery, Divine Mercy Hospital Limited, Gazipur, BGD; 2 Department of Biochemistry, Delta Medical College and Hospital, Dhaka, BGD

**Keywords:** cholesterol, csom, dyslipidemia, otitis media, recurrent otitis media

## Abstract

Background: Chronic suppurative otitis media (CSOM) is a major cause of preventable hearing loss, particularly in resource-limited, semi-urban healthcare settings where advanced radiological imaging is often unavailable for recurrence risk stratification. Systemic dyslipidemia has been mechanistically linked to mucosal inflammation and epithelial dysfunction, but its association with CSOM recurrence has not been directly examined. This study investigated the clinical utility of fasting serum lipid profiles as predictors of recurrence in patients with CSOM.

Methods: We conducted a retrospective, electronic health record-based cohort study of 160 patients diagnosed with CSOM at Mirzapur Modern Hospital, Tangail, Bangladesh, between June 2023 and May 2024, followed for 12 months post-treatment. Patients were stratified into recurrent (Group A, n = 68) and non-recurrent (Group B, n = 92) cohorts. Continuous variables were compared with independent Student's t-tests; categorical variables were compared with chi-square tests incorporating Yates' continuity correction for 2×2 tables. Multivariable binary logistic regression identified independent predictors of recurrence, and receiver operating characteristic (ROC) curve analysis with Youden's index determined optimal diagnostic cutoffs.

Results: The recurrent cohort had significantly higher mean total cholesterol (211.19 ± 24.55 vs. 183.81 ± 20.89 mg/dL), low-density lipoprotein cholesterol (LDL-C; 140.02 ± 22.51 vs. 110.71 ± 19.26 mg/dL), and triglycerides (180.26 ± 30.40 vs. 142.30 ± 28.61 mg/dL; all p < 0.001), and significantly lower high-density lipoprotein cholesterol (HDL-C; 35.12 ± 5.18 vs. 44.63 ± 5.97 mg/dL; p < 0.001) than the non-recurrent cohort. On multivariable analysis, LDL-C (adjusted odds ratio (OR) 1.088, 95% CI 1.051-1.127), HDL-C (OR 0.826, 95% CI 0.745-0.916), triglycerides (OR 1.029, 95% CI 1.009-1.049), and atticoantral (unsafe) disease type (OR 6.838, 95% CI 2.109-22.185) were independent predictors of recurrence (all p ≤ 0.005). LDL-C demonstrated the highest diagnostic accuracy (area under the curve (AUC) 0.850; optimal cutoff 138.5 mg/dL; sensitivity 64.7%, specificity 92.4%).

Conclusions: Baseline fasting lipid derangement is independently associated with CSOM recurrence, and the fasting lipid panel is a promising, low-cost candidate marker for recurrence risk stratification in resource-limited healthcare settings. As these findings derive from a retrospective, single-center cohort without external validation, the lipid panel should be regarded as hypothesis-generating rather than a validated clinical tool; prospective, multicenter studies with external validation and direct lipidomic analysis of middle ear effusion are needed before it can be positioned as a practical stratification instrument.

## Introduction

Chronic suppurative otitis media (CSOM) is a persistent inflammatory and infectious disease of the middle ear and mastoid cavity, characterized by otorrhoea through a perforated tympanic membrane for more than two to six weeks and almost invariably accompanied by conductive hearing loss. A 2025 systematic review estimated that CSOM affects approximately 3.8% of the global population, or roughly 297 million people, 85% of whom live in low- and middle-income countries; 62% of affected individuals have disabling hearing loss [1]. Earlier global burden-of-disease modelling estimated an annual CSOM incidence of 4.76 per 1,000 population and attributed approximately 21,000 deaths per year to complications of otitis media [2]. In Bangladesh, CSOM disproportionately affects rural and semi-urban populations of lower socioeconomic status, with *Staphylococcus aureus* and *Pseudomonas aeruginosa* remaining the predominant pathogens isolated in tertiary care settings [3].

Despite appropriate medical or surgical therapy, a substantial subset of patients experience recalcitrant, recurrent otorrhoea. Eustachian tube dysfunction (ETD) has recently been shown to be a powerful, quantifiable predictor of this recurrent phenotype: in a cross-sectional study of 212 recurrent chronic otitis media patients matched to controls, higher tubal opening pressures and lower Valsalva success rates independently predicted faster time to recurrence [4]. Middle ear mucosal biofilm has also been implicated in the chronicity of mucosal-type CSOM [5]. However, the pathophysiology driving recurrence remains multifactorial, and systemic metabolic status is increasingly recognized as a modifier of the local inflammatory microenvironment of the middle ear.

Acute, severe systemic inflammation produces a lipid signature of its own: total cholesterol, low-density lipoprotein cholesterol (LDL-C), and high-density lipoprotein cholesterol (HDL-C) have been shown to fall as C-reactive protein rises in acutely hospitalized patients, a pattern described as an “inflammatory lipid paradox” that reflects the hepatic acute-phase response to critical illness rather than a fixed, pre-existing metabolic state [6]. As detailed in the Discussion, this acute-phase pattern is mechanistically distinct from the chronic, stable dyslipidemia examined in the present study. Independently, high-density lipoprotein's own anti-inflammatory, endothelium-protective capacity is impaired under conditions of chronic low-grade inflammation and hyperglycemia [7]. The relationship between lipids and mucosal inflammation is also bidirectional: systemic dyslipidemia can act as a driver, rather than merely a consequence, of local mucosal inflammation. Cholesterol is a fundamental structural component of lipid rafts, cholesterol- and sphingomyelin-rich membrane microdomains that organize inflammatory signaling. A transcriptomic and functional study of CSOM granulation tissue identified marked overexpression of the lipid raft linker protein RFTN1, and showed that RFTN1 inhibition suppressed lipid raft formation, Toll-like receptor 4 signaling, and the downstream inflammatory response to *Staphylococcus aureus* and *Bacillus cereus* in both cellular and rat models of CSOM [8]. Complementary work in human lung epithelial cells has demonstrated that *S. aureus* alpha-hemolysin exploits caveolin-1- and cholesterol-rich lipid rafts to gain intracellular entry, an entry route substantially attenuated by cholesterol depletion [9]--a mechanism plausibly relevant to bacterial persistence within the middle ear mucosa.

Beyond membrane signaling, Eustachian tube mucociliary clearance depends on a surfactant layer composed predominantly of phospholipids and cholesterol together with surfactant-associated proteins SP-A, SP-B, and SP-D, which lowers tubal opening pressure and facilitates active clearance of pathogens from the middle ear [10,11]. Alterations in local phospholipid composition have historically been associated with secretory otitis media [12]. Chronic exposure to excess cholesterol has also recently been shown, in primary airway epithelial cultures, to inhibit p53 nuclear translocation and to shift epithelial differentiation away from ciliated cells toward a secretory phenotype, providing a plausible mechanistic bridge between systemic hypercholesterolemia and impaired mucociliary function [13]. Persistent negative middle ear pressure and microhemorrhage further predispose to the deposition of cholesterol crystals and the formation of cholesterol granulomas, an established histopathological correlate of recalcitrant middle ear disease [14]. At the biochemical level, the arachidonic acid cascade--initiated by phospholipase A2 cleavage of membrane phospholipids and culminating in leukotriene B4 and prostaglandin E2 production--has been shown to be a major determinant of leukocyte recruitment in experimental otitis media, and elevated middle ear fluid leukotriene B4 has previously been associated with treatment failure and recurrence in children with acute otitis media [15]. Circulating LDL-C is also susceptible to oxidative modification; oxidized LDL binding to the scavenger receptor lectin-like oxidized low-density lipoprotein receptor-1 (LOX-1) amplifies inflammatory signaling and has been demonstrated experimentally in models of bacterial endotoxemia [16,17], while HDL-C ordinarily counteracts this process through its antioxidant and reverse cholesterol transport functions.

Serum lipid abnormalities have previously and consistently been linked to sensorineural hearing loss through microvascular and cochlear mechanisms [18-20], but their association with CSOM recurrence specifically has not been directly investigated. The closest existing evidence derives from studies of oxidative stress in chronic otitis media, which have demonstrated elevated serum markers of lipid peroxidation and reduced antioxidant capacity in affected patients [21,22], and from pediatric studies linking elevated serum total cholesterol to otitis media with effusion [23]. A contrasting view, articulated by Sadé and Teitz, holds that cholesterol accumulating within middle ear effusions and cholesteatomas is generated locally by tissue breakdown rather than derived from the circulation [24]; our hypothesis concerns the systemic, not local, effects of circulating lipids on mucosal immunity and epithelial function, and does not depend on serum cholesterol depositing directly within the middle ear. Given the cost and logistical barriers to advanced imaging in semi-urban healthcare settings, identifying an inexpensive, routinely available biochemical marker of recurrence risk would be of considerable clinical value. We therefore investigated the association between fasting serum lipid profile parameters and CSOM recurrence using retrospective electronic health records from a semi-urban hospital in Tangail, Bangladesh.

## Materials and methods

Study design and clinical setting

This retrospective, single-center, electronic health record-based cohort study evaluated the relationship between fasting serum lipid profile parameters and disease recurrence in patients diagnosed with CSOM at Mirzapur Modern Hospital, Tangail, Bangladesh, a private secondary care facility serving a semi-urban and rural catchment area within the Dhaka division. Records from patients managed between June 2023 and May 2024 were analyzed.

Selection of participants and sample size

A total of 160 patients diagnosed with CSOM who met the predefined eligibility criteria were included, representing the entire eligible patient population retrieved from the hospital's electronic health record database within the 12-month study window. Inclusion criteria were: (1) a confirmed clinical diagnosis of CSOM, defined as persistent or intermittent otorrhoea lasting at least two weeks with a permanent tympanic membrane perforation on otoscopy; (2) a complete fasting serum lipid profile documented at initial presentation or pre-operative screening prior to definitive treatment; (3) complete baseline clinical data including CSOM type (safe/tubotympanic vs. unsafe/atticoantral) and treatment modality; and (4) documented continuous follow-up of at least 12 months post-treatment. Patients were excluded for current or recent (within six months) lipid-lowering pharmacotherapy; conditions causing secondary dyslipidemia (hypothyroidism, nephrotic syndrome, chronic kidney disease, severe hepatic dysfunction, uncontrolled diabetes mellitus); concomitant systemic inflammatory or autoimmune disease; craniofacial or ciliary dyskinesia syndromes; pregnancy or lactation; or incomplete records.

Clinical management and group allocation

Patients were stratified into Group A (recurrent CSOM, n = 68): two or more documented episodes of active purulent otorrhoea within 12 months of primary therapy, or persistent otorrhoea unresponsive to compliant medical or surgical treatment requiring secondary intervention; and Group B (non-recurrent CSOM, n = 92): complete resolution (“dry ear”) maintained throughout 12 months of follow-up. Primary treatment was categorized as medical management (aural toileting with topical antiseptic or quinolone antibiotic drops for 7-14 days) or surgical management (myringoplasty, tympanoplasty, or mastoidectomy).

Extraction of clinical and biochemical data

A standardized digital data collection sheet was used to extract age, sex, smoking history, controlled type 2 diabetes status, CSOM type, and treatment modality. Fasting serum lipid profiles were drawn after a 12-hour overnight fast. Total cholesterol was measured by the cholesterol oxidase-peroxidase aminoantipyrine phenol (CHOD-PAP) enzymatic colorimetric assay, triglycerides by the glycerol phosphate oxidase-peroxidase aminoantipyrine phenol (GPO-PAP) method, and HDL-C by a direct homogeneous enzymatic colorimetric method. Very-low-density lipoprotein cholesterol (VLDL-C) was calculated as triglycerides/5, and LDL-C was calculated using the Friedewald formula (LDL-C = total cholesterol − HDL-C − VLDL-C); this formula was not applied to any sample with triglycerides exceeding 400 mg/dL, above which its assumptions are known to be invalid.

Statistical analysis

Analyses were performed using SciPy and Statsmodels in Python (Python Software Foundation, Wilmington, DE, USA). Continuous variables were assessed for normality using the Shapiro-Wilk test; as all conformed to a normal distribution, they are expressed as mean ± standard deviation and were compared between groups using the independent Student's t-test. Categorical variables are presented as frequencies and percentages and were compared using the chi-square test of contingency with Yates' continuity correction for 2×2 tables. Multivariable binary logistic regression was used to identify independent biochemical predictors of recurrence (dependent variable: 0 = non-recurrent, 1 = recurrent) after adjustment for age, sex, smoking history, diabetes status, CSOM disease type, and fasting LDL-C, HDL-C, and triglycerides; adjusted odds ratios (OR) with 95% confidence intervals (CI) and Wald z-statistic p-values were calculated. Total cholesterol and VLDL-C were not entered into the multivariable model because both are algebraically derived from the three included lipid components (total cholesterol = LDL-C + HDL-C + VLDL-C; VLDL-C = triglycerides/5), and their inclusion alongside LDL-C, HDL-C, and triglycerides would have produced perfect collinearity; both parameters were instead evaluated separately by univariate comparison and receiver operating characteristic (ROC) analysis. Overall model performance was summarized using the pseudo-R² and likelihood-ratio test reported in Results. ROC curve analysis was conducted to evaluate the diagnostic performance of total cholesterol, LDL-C, HDL-C, and triglycerides, with the area under the curve (AUC) calculated with 95% CI. Optimal cutoff values were determined using Youden's index (J = sensitivity + specificity − 1) [25], which identifies the cutoff maximizing the vertical distance between the ROC curve and the diagonal reference line under the implicit assumption of equal misclassification cost [26]. A two-tailed p-value <0.05 was considered statistically significant throughout.

## Results

Demographic and clinical characteristics

Of 160 patients with CSOM followed over 12 months, 68 (42.5%) experienced recurrence (Group A) and 92 (57.5%) remained recurrence-free (Group B). Table 1 compares baseline demographic and clinical characteristics between cohorts. There were no significant differences between groups in mean age (36.81 ± 9.78 vs. 36.33 ± 11.81 years; p = 0.785), sex distribution (p = 0.176), diabetes prevalence (p = 0.135), smoking history (p = 0.519), or treatment modality (p = 0.980). A highly significant difference was observed in CSOM type: 60.3% of the recurrent cohort had atticoantral (unsafe) disease compared with 19.6% of the non-recurrent cohort (p < 0.001).

**Table 1:** Comparison of baseline demographic and clinical characteristics between the recurrent and non-recurrent CSOM cohorts (n = 160). ᵃChi-square statistic calculated with Yates' continuity correction for 2×2 contingency tables. CSOM: chronic suppurative otitis media; T2DM: type 2 diabetes mellitus.

Variable	Total cohort (n = 160)	Recurrent Group A (n = 68)	Non-recurrent Group B (n = 92)	Test statistic (χ²/t)	p-value
Age (years), mean ± SD	36.53 ± 11.01	36.81 ± 9.78	36.33 ± 11.81	t = 0.273	0.785
Male sex, n (%)	77 (48.1)	28 (41.2)	49 (53.3)	χ² = 1.829ᵃ	0.176
Female sex, n (%)	83 (51.9)	40 (58.8)	43 (46.7)	-	-
Diabetic (controlled T2DM), n (%)	26 (16.2)	15 (22.1)	11 (12.0)	χ² = 2.237ᵃ	0.135
Non-diabetic, n (%)	134 (83.8)	53 (77.9)	81 (88.0)	-	-
Smoker, n (%)	44 (27.5)	21 (30.9)	23 (25.0)	χ² = 0.416ᵃ	0.519
Non-smoker, n (%)	116 (72.5)	47 (69.1)	69 (75.0)	-	-
Atticoantral (unsafe) CSOM, n (%)	59 (36.9)	41 (60.3)	18 (19.6)	χ² = 26.142ᵃ	<0.001
Tubotympanic (safe) CSOM, n (%)	101 (63.1)	27 (39.7)	74 (80.4)	-	-
Surgical treatment, n (%)	101 (63.1)	43 (63.2)	58 (63.0)	χ² = 0.001ᵃ	0.980
Medical treatment, n (%)	59 (36.9)	25 (36.8)	34 (37.0)	-	-

Comparison of fasting serum lipid profiles

**Figure 1:**
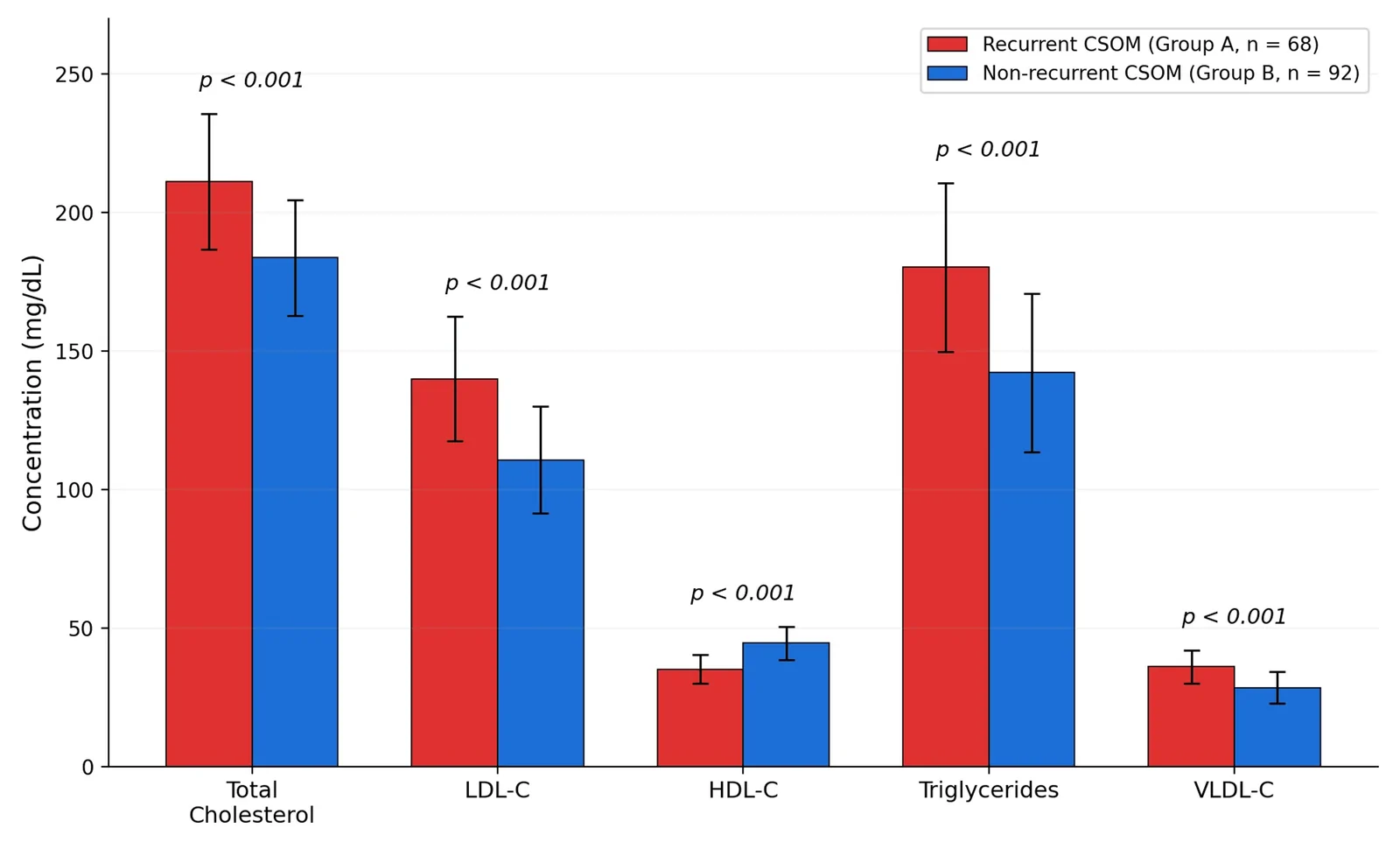
Comparison of fasting serum lipid parameters (mean ± SD) between recurrent (Group A, n = 68) and non-recurrent (Group B, n = 92) CSOM cohorts. All comparisons p < 0.001 by independent Student's t-test. CSOM: chronic suppurative otitis media; LDL-C: low-density lipoprotein cholesterol; HDL-C: high-density lipoprotein cholesterol; VLDL-C: very-low-density lipoprotein cholesterol.

All fasting lipid parameters were significantly deranged in the recurrent cohort (Table 2, Figure 1). Mean total cholesterol (211.19 ± 24.55 vs. 183.81 ± 20.89 mg/dL), LDL-C (140.02 ± 22.51 vs. 110.71 ± 19.26 mg/dL), triglycerides (180.26 ± 30.40 vs. 142.30 ± 28.61 mg/dL), and calculated VLDL-C (36.05 ± 6.08 vs. 28.47 ± 5.73 mg/dL) were all significantly higher in Group A than Group B (all p < 0.001). Conversely, HDL-C was significantly lower in the recurrent cohort (35.12 ± 5.18 vs. 44.63 ± 5.97 mg/dL; p < 0.001).

**Table 2:** Comparison of fasting serum lipid profiles between the recurrent and non-recurrent CSOM cohorts. CSOM: chronic suppurative otitis media; LDL: low-density lipoprotein; HDL: high-density lipoprotein; VLDL: very-low-density lipoprotein.

Lipid parameter (mg/dL)	Recurrent Group A (n = 68)	Non-recurrent Group B (n = 92)	t-statistic	p-value
Total cholesterol	211.19 ± 24.55	183.81 ± 20.89	7.60	<0.001
LDL-cholesterol	140.02 ± 22.51	110.71 ± 19.26	8.85	<0.001
HDL-cholesterol	35.12 ± 5.18	44.63 ± 5.97	-10.53	<0.001
Triglycerides	180.26 ± 30.40	142.30 ± 28.61	8.08	<0.001
VLDL-cholesterol	36.05 ± 6.08	28.47 ± 5.73	8.06	<0.001

Multivariable logistic regression analysis

The multivariable logistic regression model, adjusting for age, sex, smoking, diabetes status, and CSOM type, demonstrated strong predictive performance (pseudo-R² = 0.595; likelihood-ratio p < 0.001). Table 3 details the full model. Each 1 mg/dL increase in fasting LDL-C was independently associated with an 8.8% increase in the odds of recurrence (adjusted OR 1.088, 95% CI 1.051-1.127; p < 0.001), while each 1 mg/dL increase in HDL-C was associated with a 17.4% reduction in the odds of recurrence (adjusted OR 0.826, 95% CI 0.745-0.916; p < 0.001). Triglycerides showed a smaller but significant independent association (adjusted OR 1.029, 95% CI 1.009-1.049; p = 0.005). Atticoantral disease type carried a nearly seven-fold increase in the adjusted odds of recurrence relative to tubotympanic disease (adjusted OR 6.838, 95% CI 2.109-22.185; p = 0.001). Age, sex, diabetes status, and smoking were not independently associated with recurrence in the adjusted model.

**Table 3:** Multivariable binary logistic regression model of clinical and biochemical predictors of CSOM recurrence. Wald z-statistics were recalculated as β/SE for internal consistency with the reported coefficients; this recalculation did not change the direction, magnitude, or statistical significance of any association. CSOM: chronic suppurative otitis media; LDL: low-density lipoprotein; HDL: high-density lipoprotein.

Predictor	β	SE	Wald z	Adjusted OR	95% CI	p-value
Intercept	-8.263	3.536	-2.337	0.0003	-	0.019
Age, years	-0.014	0.028	-0.500	0.986	0.935-1.041	0.617
Sex (male vs. female)	-0.703	0.566	-1.242	0.495	0.163-1.500	0.214
Diabetes (yes vs. no)	0.688	0.690	0.997	1.989	0.514-7.698	0.319
Smoking (yes vs. no)	0.206	0.604	0.341	1.229	0.376-4.011	0.733
CSOM type (atticoantral)	1.923	0.600	3.205	6.838	2.109-22.185	0.001
LDL-cholesterol, mg/dL	0.085	0.018	4.722	1.088	1.051-1.127	<0.001
HDL-cholesterol, mg/dL	-0.191	0.053	-3.604	0.826	0.745-0.916	<0.001
Triglycerides, mg/dL	0.028	0.010	2.800	1.029	1.009-1.049	0.005

Diagnostic performance of lipid parameters

**Figure 2:**
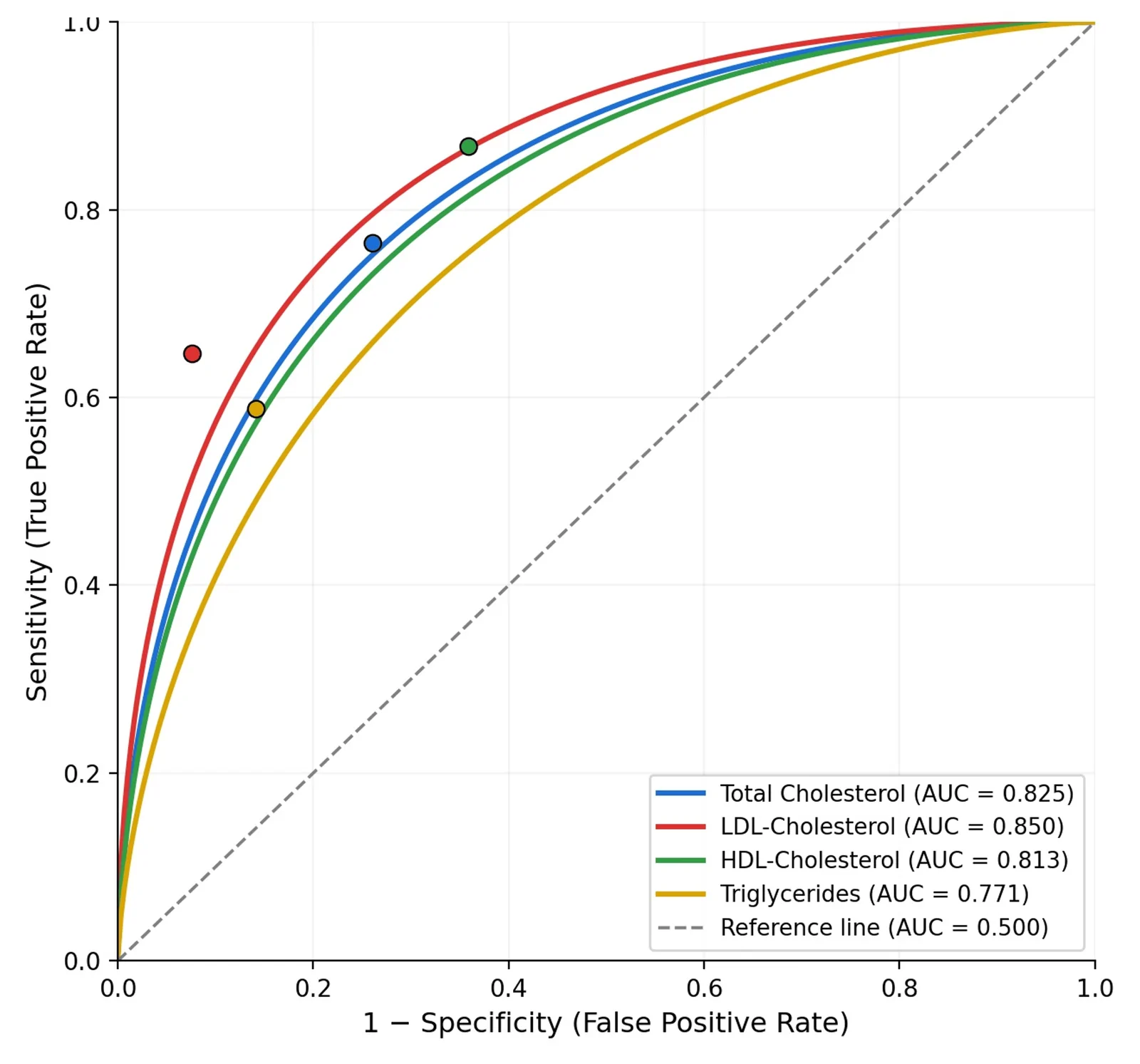
Receiver operating characteristic (ROC) curves for fasting total cholesterol, LDL-cholesterol, HDL-cholesterol, and triglycerides in predicting CSOM recurrence. Curves are reconstructed from the reported area under the curve using a binormal model; marked points indicate the Youden-optimal sensitivity/specificity operating point for each parameter (Table 4). CSOM: chronic suppurative otitis media; LDL: low-density lipoprotein; HDL: high-density lipoprotein; AUC: area under the curve.

ROC curve analysis (Table 4, Figure 2) demonstrated that baseline LDL-C had the strongest discriminative performance for CSOM recurrence within this cohort (AUC 0.850, 95% CI 0.789-0.911). At the Youden-optimal cutoff of 138.5 mg/dL, LDL-C predicted recurrence with 64.7% sensitivity and 92.4% specificity (J = 0.571). Total cholesterol (AUC 0.825) and HDL-C (AUC 0.813) also demonstrated good discriminative performance. Because HDL-C is inversely related to recurrence risk, its optimal cutoff of 41.3 mg/dL represents a lower threshold below which risk increases (sensitivity 86.8%, specificity 64.1%). Triglycerides showed the lowest, though still acceptable, discriminative performance (AUC 0.771).

**Table 4:** ROC curve metrics of fasting serum lipid fractions for prediction of CSOM recurrence. CSOM: chronic suppurative otitis media; ROC: receiver operating characteristic; LDL: low-density lipoprotein; HDL: high-density lipoprotein; AUC: area under the curve.

Lipid parameter	AUC (95% CI)	Optimal cutoff (mg/dL)	Sensitivity (%)	Specificity (%)	Youden's J
Total cholesterol	0.825 (0.758-0.892)	198.30	76.47	73.91	0.504
LDL-cholesterol	0.850 (0.789-0.911)	138.50	64.71	92.39	0.571
HDL-cholesterol	0.813 (0.744-0.881)	≤41.30	86.76	64.13	0.509
Triglycerides	0.771 (0.697-0.844)	168.90	58.82	85.87	0.447

## Discussion

This retrospective cohort study shows that fasting serum lipid derangement--elevated total cholesterol, LDL-C, and triglycerides alongside depleted HDL-C--is strongly and independently associated with CSOM recurrence, even after adjustment for established clinical risk factors, including atticoantral disease type. As far as we are aware, no earlier study has directly compared a full serum lipid panel between patients with and without CSOM recurrence. The closest available evidence comes from work demonstrating elevated serum markers of oxidative stress and lipid peroxidation, together with reduced antioxidant and paraoxonase/arylesterase activity, in patients with chronic otitis media [21,22], and from a pediatric case-control study linking elevated serum total cholesterol to otitis media with effusion [23]. Our findings extend this literature by describing a specific, quantifiable, and clinically actionable association between the fasting lipid panel and CSOM recurrence in an adult, semi-urban South Asian cohort.

This directionality is worth reconciling explicitly with the acute-phase “inflammatory lipid paradox,” in which total cholesterol, LDL-C, and HDL-C fall as C-reactive protein rises in acutely hospitalized patients [6]. That phenomenon reflects a short-term hepatic acute-phase response--altered lipoprotein synthesis, clearance, and redistribution during severe systemic illness or critical illness-- and is mechanistically and temporally distinct from the pattern observed in our cohort. Lipid panels in the present study were drawn from clinically stable outpatients at initial presentation or pre-operative screening, not during an episode of acute systemic illness, sepsis, or hospitalization for critical illness, and patients with overt secondary causes of dyslipidemia or concurrent systemic inflammatory disease were excluded by design. In this setting, the elevated LDL-C and triglycerides and depleted HDL-C we observed most plausibly reflect a pre-existing, chronic atherogenic lipid phenotype that predates and predisposes to recurrent local mucosal inflammation, rather than a lipid profile acutely suppressed by the CSOM episode itself. The direction of the relationship between systemic inflammation and circulating lipids therefore appears to depend heavily on the acuity and severity of the inflammatory stimulus: transient suppression during acute critical illness versus a stable, elevated atherogenic profile that may causally contribute to the chronic, low-grade mucosal inflammation implicated in CSOM recurrence.

A few further biologically plausible pathways could link chronic systemic dyslipidemia to CSOM recurrence, although none has yet been tested directly in ear tissue. Cholesterol is a core structural component of lipid rafts, and CSOM granulation tissue has been shown to overexpress the raft-organizing protein RFTN1; inhibiting RFTN1 reduces lipid raft formation, Toll-like receptor 4 recruitment, and downstream inflammatory cytokine production in both cellular and animal models of CSOM [8]. Because raft assembly depends on an adequate membrane cholesterol pool, higher circulating LDL-C could plausibly enlarge the substrate available for raft-mediated inflammatory signaling in the middle ear epithelium. Cholesterol-rich lipid rafts have also been shown to mediate the intracellular entry of *Staphylococcus aureus* into respiratory epithelial cells--an entry route substantially reduced by cholesterol depletion [9]--raising the possibility that systemic hypercholesterolemia facilitates bacterial persistence and recurrent infection through a related route.

A related but distinct pathway concerns epithelial differentiation itself. Chronic hypercholesterolemia has recently been shown, in primary human bronchial epithelial cultures, to inhibit p53 nuclear translocation and to shift epithelial differentiation toward a secretory phenotype at the expense of ciliated cells [13]. Because Eustachian tube and middle ear mucociliary clearance depend on an intact ciliated epithelium together with a surfactant layer rich in phospholipids and cholesterol [10-12], a cholesterol-driven fall in ciliated cell density offers a plausible structural explanation for impaired clearance, mucus stasis, and recurrent bacterial colonization in patients with elevated LDL-C.

At the biochemical level, the arachidonic acid-eicosanoid cascade--triggered by phospholipase A2 cleavage of membrane phospholipids--is a major driver of leukocyte recruitment in experimental otitis media, and elevated middle ear leukotriene B4 has previously been linked to treatment failure in acute otitis media [15]. Sustained elevation of circulating triglycerides and cholesterol may expand the membrane phospholipid pool available to phospholipase A2, prolonging eicosanoid-driven inflammation and slowing mucosal healing. Separately, oxidative modification of circulating LDL-C into oxidized LDL, which signals through the scavenger receptor LOX-1, has been demonstrated experimentally under conditions of bacterial endotoxemia [16,17]; HDL-C ordinarily limits this process through its antioxidant and reverse cholesterol transport activity, a protective function that is itself impaired by chronic low-grade inflammation and hyperglycemia [7]. The pattern observed in our cohort--elevated LDL-C together with depleted HDL-C--is therefore consistent with a pro-oxidative, pro-inflammatory systemic lipid state that could aggravate local mucosal injury while simultaneously weakening antioxidant defenses.

A longstanding and different view, put forward by Sadé and Teitz, holds that the cholesterol found within middle ear effusions and cholesteatomas is generated locally through tissue and erythrocyte breakdown rather than derived directly from the circulation, based on a cholesterol-to-cholesterol-ester ratio in effusions that differed from that of serum [24]. Our findings do not require serum cholesterol to deposit directly within the middle ear to explain its association with recurrence; rather, the mechanisms proposed above act at the level of systemic effects on epithelial differentiation, membrane signaling, and circulating inflammatory or oxidative mediators, which in turn influence the local mucosal environment. The two views are complementary rather than competing: local cholesterol deposition may still arise mainly from microhemorrhage and cellular breakdown, while systemic dyslipidemia independently shapes the inflammatory and structural substrate on which that local process unfolds.

From a practical standpoint, these findings suggest that a fasting lipid panel--a routine, inexpensive test often already obtained as part of pre-operative anesthesia screening--could assist risk stratification in semi-urban and rural healthcare settings where high-resolution temporal bone imaging is often unavailable. Patients with baseline LDL-C above 138.5 mg/dL or HDL-C below 41.3 mg/dL might be flagged as being at elevated risk of recurrence, potentially informing a more aggressive initial surgical strategy, closer post-treatment follow-up, and adjunctive counselling on dietary and lifestyle modification of dyslipidemia. These proposed cutoffs, however, are derived from a single retrospective cohort and have not been externally validated; they should be regarded as hypothesis-generating rather than as ready-to-use clinical thresholds until confirmed prospectively in independent, multicenter populations.

This study has several limitations. Its retrospective design precludes causal inference between dyslipidemia and CSOM recurrence, and the proposed diagnostic cutoffs have not been externally validated in an independent cohort. As a single-center study from a semi-urban hospital in Tangail, Bangladesh, findings may not generalize to urban populations or to other geographic and dietary contexts. Lipid panels were drawn at initial presentation or pre-operative screening rather than at a uniformly standardized time point relative to disease onset, which may introduce some temporal heterogeneity. As with any observational study, residual confounding from variables not captured in the electronic health record cannot be excluded; in particular, we did not capture granular measures of baseline disease severity (such as extent of mucosal disease, granulation tissue, or prior surgical history), post-treatment adherence to prescribed aural care and antibiotic regimens, or socioeconomic factors including household income, nutritional status, and access to follow-up care, all of which plausibly influence both lipid metabolism and CSOM recurrence risk independently of the associations reported here. We also did not formally test the multivariable model's collinearity structure or calibration beyond excluding total cholesterol and VLDL-C for the exact algebraic collinearity described in Methods; variance inflation factor diagnostics and a formal goodness-of-fit test (e.g., the Hosmer-Lemeshow test) were not performed, and future prospective work should incorporate these checks explicitly. Finally, because of resource constraints, we did not directly assay local middle ear effusion lipid or lipidomic composition, oxidative stress markers, or inflammatory cytokines, which would be needed to establish the mechanistic pathways discussed above. Prospective, multicenter studies incorporating external validation and direct lipidomic analysis of middle ear fluid are needed to clarify the cellular and molecular mechanisms linking systemic lipid metabolism to middle ear mucosal immunity and to confirm the clinical utility of the proposed cutoffs in independent cohorts.

## Conclusions

In this retrospective cohort of 160 patients with chronic suppurative otitis media, fasting serum LDL-C, HDL-C, and triglycerides were independent predictors of 12-month recurrence, and LDL-C in particular showed the strongest discriminative performance (AUC 0.850) at a preliminary cutoff of 138.5 mg/dL. These findings should be regarded as hypothesis-generating: given the retrospective, single-center design and the absence of external validation, the fasting lipid panel is best characterized as a promising, low-cost candidate marker rather than a validated clinical tool. Prospective, multicenter studies with external validation are needed before it can be adopted as a practical risk-stratification instrument for CSOM recurrence in otolaryngology practice.
